# Reply to Veerman et al. Comment on “Rosenzweig et al. Very Low Prostate PET/CT PSMA Uptake May Be Misleading in Staging Radical Prostatectomy Candidates. *J. Pers. Med.* 2022, *12*, 410”

**DOI:** 10.3390/jpm12060861

**Published:** 2022-05-25

**Authors:** Barak Rosenzweig, Rennen Haramaty, Tima Davidson, Alon Lazarovich, Asaf Shvero, Miki Haifler, Jonathan Gal, Shay Golan, Sagi Shpitzer, Azik Hoffman, Omri Nativ, Yuval Freifeld, Rani Zreik, Zohar A. Dotan

**Affiliations:** 1 Department of Urology, Chaim Sheba Medical Center, Ramat Gan 5211401, Israel; rennen.haramaty@sheba.health.gov.il (R.H.); alon.lazarovich@sheba.health.gov.il (A.L.); asaf.shvero@sheba.health.gov.il (A.S.); zohar.dotan@sheba.health.gov.il (Z.A.D.); 2 Sackler Faculty of Medicine, Tel Aviv University, Tel Aviv 6997801, Israel; tima.davidson@sheba.health.gov.il (T.D.); michael.haifler@sheba.health.gov.il (M.H.); shaygo1@gmail.com (S.G.); 3 Department of Nuclear Medicine, Chaim Sheba Medical Center, Ramat Gan 5211401, Israel; 4 Department of Urology, Shamir Medical Center, Tzrifin 6093000, Israel; jonathan.gal8@gmail.com; 5 Section of Urology, Rabin Medical Center, Petah Tikva 4941492, Israel; sagi.shpitzer@gmail.com; 6 Department of Urology, Rambam Health Center, Haifa 3109601, Israel; azikhof@gmail.com (A.H.); o_nativ@rambam.health.gov.il (O.N.); 7 Ruth and Bruce Rappaport Faculty of Medicine, Technion Israel Institute of Technology, Haifa 3200003, Israel; yuvalfrei@gmail.com (Y.F.); ranizreik@gmail.com (R.Z.); 8 Department of Urology, Carmel Medical Center, Haifa 3436212, Israel

We thank the commenters for their important insights [1]. In our work, we chose to interchangeably use the terms “very low prostate PET/CT PSMA uptake” (title) and “negative” (using a quotation mark). Although semantics sometimes represent true critical differences, we believe the current phrasing, especially when applied in the preoperative setting, represents a similar concept, i.e., non-suspected prostate. As our goal was to emphasize the surgeon’s clinical application of PET/CT PSMA, we believe these phrasings represent a “real-life” perspective. 

Regarding SUVmax, we completely agree with the commenters and find this cutoff to represent an important part of the study methods. Looking into several examples in the field, Ruschoff et al. suggested a normal SUVmax value to range from 3.15 to 9.1, while Emmet et al. regarded different SUVmax cutoff values in their supplementary data, defining SUVmax specificity to range between 84% and 94% for values between 6 and 7, respectively, representing ~88% specificity for the value we applied (6.6) [2,3]. In our work, we accordingly chose a SUVmax cutoff in the “middle ground”, based upon Uprimny et al. defining the normal SUVmax cutoff as 6.6 [4]. Such variability is not uncommon in academic publications. As mentioned in our study limitations, the data are subject to variability having been gathered from five medical institutes subjects; however, such team effort was necessitated in order to assemble a large enough cohort of patients who complied with our study inclusion criteria. The fact that radiologists and pathologists in all of the participating medical centers are highly experienced and dedicated professionals may partially compensate for this effect. 

Our data exemplify a high acceptance rate for PET/CT PSMA usage in the preoperative setting. This represents agreement with PSMA uptake to correlate with prostate cancer aggressiveness. However, the current work suggests that a subpopulation of patients with clinically significant cancer and aggressive characteristics show deceptively weak PSMA uptake. Although prostate-specific membrane antigen (PSMA), as its name implies, was initially suggested as an highly specific tracer, as data were gathered, its specificity limitation was acknowledged [5,6]. Considering the fact that PSMA PET/CT is now suggested as a suitable replacement for conventional imaging by providing superior accuracy for staging patients with high-risk prostate cancer before curative-intent treatment [7], we believe similar questions regarding its ability in this clinical setting should be noted. Finding very low/“negative” PET/CT PSMA reads amongst patients encompassing higher-risk disease supports this hypothesis.

We again wish to thank our commenters for raising this discussion. We believe such discussions will lead our community to recognize the true benefit as well as limitations of any new technology we adopt. We will of course be happy to share future oncological data of this cohort when the time comes.

## Data Availability

The data presented in this study are available on request from the corresponding author. The data are not publicly available due to confidentiality of medical records.
